# Quantitative Ultrasonographic Analysis of Changes of the Suprascapular Nerve in the Aging Population With Shoulder Pain

**DOI:** 10.3389/fbioe.2021.640747

**Published:** 2021-02-19

**Authors:** Wei-Ting Wu, Lan-Rong Chen, Hsiang-Chi Chang, Ke-Vin Chang, Levent Özçakar

**Affiliations:** ^1^Department of Physical Medicine and Rehabilitation, National Taiwan University Hospital, Bei-Hu Branch, Taipei, Taiwan; ^2^Department of Physical Medicine and Rehabilitation, Cheng Hsin General Hospital, Taipei, Taiwan; ^3^Department of Physical Medicine and Rehabilitation, National Taiwan University College of Medicine, Taipei, Taiwan; ^4^Department of Physical and Rehabilitation Medicine, Hacettepe University Medical School, Ankara, Turkey

**Keywords:** aging, rotator cuff, degeneration, neuropathy, ultrasonography

## Abstract

**Background:**

Older people are vulnerable to painful shoulder syndromes, the majority of which are derived from degenerative rotator cuff pathologies. The suprascapular nerve (SSN) is closely related to the rotator cuff complex, and its role in shoulder pain has recently been highlighted. This study aimed to explore the differences in SSN among older people with and without shoulder pain, and to investigate the potential factors influencing the nerve size using ultrasound (US) imaging.

**Methods:**

Participants aged ≥60 years were enrolled in the study. A systematic and bilateral US examination of the rotator cuff tendons was performed. The SSN was examined from its origin in the brachial plexus to the spinoglenoid notch of the infraspinatus fossa. The association between the nerve’s cross-sectional area (CSA) and rotator cuff lesions was analyzed using the generalized estimation equation.

**Results:**

Among the 94 participants, 45 (with bilaterally asymptomatic shoulders) were classified into the control group, whereas 49 (with at least one-sided shoulder pain) were classified into the group with shoulder pain. The average CSAs of the SSN at the level of the brachial plexus, supraspinatus fossa, and infraspinatus fossa were comparable between the patients in the control group and those with shoulder pain. There was a higher prevalence of rotator cuff lesions and enlarged distal SSNs in the painful shoulders than in the asymptomatic shoulders of patients with unilateral involvement. A full-thickness tear of the supraspinatus tendon was associated with swelling of the SSN in the supraspinatus fossa (β coefficient = 4.068 mm^2^, *p* < 0.001).

**Conclusion:**

In the older population, full-thickness tears of the supraspinatus tendon are independently associated with enlargement of the distal SSN. In cases with large rotator cuff tendon tears with poor response to conservative treatments, possible SSN entrapment should be considered and managed accordingly.

## Introduction

Shoulder pain is one of the most common musculoskeletal complaints, and its lifetime prevalence ranges between 6.7 and 66.7% in the general population (Luime et al., 2004). When compared with young adults, older individuals are more vulnerable to painful shoulder syndromes, with a prevalence of 41.9%, according to a prospective cohort study that enrolled 384 subjects (Imagama et al., 2020). The detrimental effect of shoulder pain on the mental health of older adults has been documented in a large-scale cross-sectional study (Rashedi et al., 2017), indicating an independent reverse association between psychological well-being and shoulder pain.

The mechanism leading to an increased risk of developing a painful shoulder with advancing age is multifactorial and includes dysfunction of the scapular muscles and degeneration of the rotator cuff tendons. Morise et al. (2017) investigated the electromyographic activity and muscle thickness of the trapezius, deltoid, and serratus anterior muscles in 45 asymptomatic subjects and found that the lower trapezius muscles were significantly thinner in the older adults than in the younger adults. A previous review by Keener et al. (2019) pointed out that poor vascularity of the rotator crescent might contribute to degenerative rotator cuff tendon tears.

The role of the suprascapular nerve (SSN) in the development and management of shoulder pain has recently been highlighted. According to an anatomic study on 17 cadavers, the SSN provides the majority of the innervation to the anterior and the superoposterior aspects of the glenohumeral joint (Eckmann et al., 2017). A previous meta-analysis indicated that the SSN block was more effective in reducing chronic shoulder pain than physical therapy and placebo injections (Chang et al., 2016). A retrospective cohort study of elite overhead athletes with rotator cuff tears revealed that arthroscopy with additional SSN decompression yielded a superior outcome than arthroscopy alone (Tsikouris et al., 2018). This study comprehensively sheds light on the role of SSN entrapment in chronic shoulder pain and dysfunction.

In recent years, high-resolution ultrasound (US) has been widely applied for the evaluation of entrapment neuropathies such as carpal and cubital tunnel syndromes. Likewise, its reliability in assessing the cross-sectional area (CSA) of the SSN has been validated by an antecedent study that enrolled 60 asymptomatic volunteers (Chang et al., 2016). US imaging of the SSN has also been shown to act as a simple surrogate marker in the diagnosis of neuralgic amyotrophy (Gruber et al., 2017). As the SSN provides motor and sensory innervation to the supraspinatus and infraspinatus muscles, it is clinically important to know whether it also plays a role in the increased prevalence of shoulder pain in the aging population. Accordingly, the purpose of the present study was two-fold. First, we aimed to explore the differences in SSN morphology between older people with and without shoulder pain. Second, using high-resolution US imaging, we aimed to identify the potential factors that might influence the nerve size.

## Materials and Methods

### Design and Participants

This was a cross-sectional study investigating rotator cuff tendon lesions and SSN in the aging population (age ≥60 years). All participants were either those who were visiting the department of physical medicine and rehabilitation for shoulder problems or those attending the geriatric clinic for the annual health examination in a community hospital in Taipei, Taiwan. Participants who were able to ambulate independently, had normal cognitive function, and were able to complete the given questionnaire were included in the study. The exclusion criteria included a history of malignancy, uncontrolled medical conditions (e.g., systemic infection and unstable angina), and previous injections or surgeries on the shoulders of either side within the last 6 months. The study was approved by the Institutional Review Board of the National Taiwan University Hospital (IRB No. 20180405RIND). All subjects provided written informed consent before joining the research project.

### Clinical Evaluation

Each participant was asked to complete the same questionnaire twice (one for each shoulder). The questionnaire was based on the Chinese version of the Shoulder Pain and Disability Index (SPADI) (Yao et al., 2017) and consisted of 13 questions categorized in two domains, pain and disability. They were instructed to indicate the degree of severity of their symptoms on a 10-point visual analog scale (VAS) for each question with a rating from 0 (no pain or difficulty) to 10 (extreme pain or difficulty). Scores from the pain and disability domains were averaged to generate the total score of SPADI, with the highest value of 100 points. Furthermore, the duration of pain and the VAS score of pain in the affected shoulders for specific situations (at rest, at night, and during overhead activity) were recorded. All participants were also evaluated bilaterally for the following: the bicipital groove tenderness and Speed, Yergason’s, empty can, Neer, Hawkins-Kennedy, and painful arc tests (Chang et al., 2020a).

### Shoulder US Examination

All participants were seated during the examination, which was conducted by a physiatrist with 12 years of musculoskeletal US experience using a linear probe of 5–18 MHz (HI VISION Ascendus, Hitachi). The transducer was placed at the level of the coracoid process to evaluate the long head of the biceps tendon inside the bicipital groove. A pathological biceps peritendinous effusion was defined when the thickness of the anechoic fluid exceeded 1 mm. Biceps tendon tear was confirmed if the tendon was invisible or split. The shoulder was then externally rotated to expose the subscapularis tendon. The supraspinatus tendon was investigated in the Middleton position (Lee et al., 2016) with the hand placed over the ipsilateral iliac crest. The transducer was finally moved to the posterior shoulder in the horizontal plane slightly distal to the scapular spine for visualization of the infraspinatus tendon. The presence of visible gaps or a total absence of tendon tissue in the subacromial space served as the criteria for diagnosing rotator cuff tendon tears. Because the supraspinatus tendon has a large size, its lesions were classified as either full-thickness or partial-thickness tears. A full-thickness tear was indicated by an intra-tendinous gap extending through the entire thickness of the supraspinatus tendon. A partial-thickness tear was indicated by noticeable intra-tendinous cleavage(s) without the involvement of the entire thickness of the tendon. All scanning procedures were performed in accordance with the EURO-MUSCULUS/USPRM basic scanning protocol for the shoulder (Ozcakar et al., 2015).

### US Imaging and Measurement of the SSN

**FIGURE 1:**
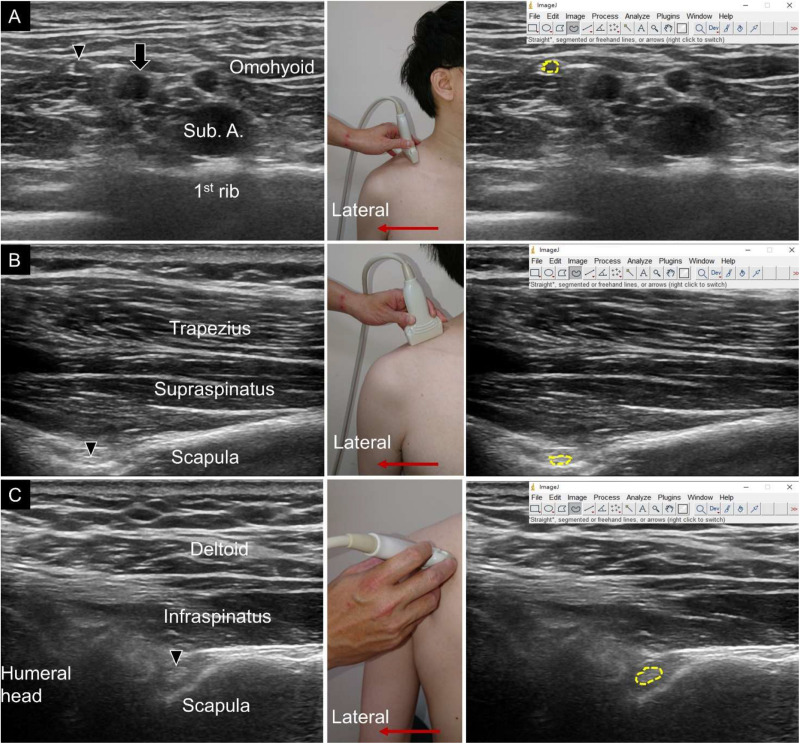
Ultrasound imaging (left subgraph), transducer placement (middle subgraph), and cross-sectional area measurement (right subgraph) for the suprascapular nerve near the brachial plexus **(A)**, at the supraspinatus fossa **(B)** and at the infraspinatus fossa **(C)**. Suprascapular nerve, black arrowheads; brachial plexus, black arrow; Sub. A, subclavian artery.

The transducer was placed over the lower cervical region to visualize the brachial plexus interposed between the anterior and middle scalene muscles. The transducer was moved more laterally to see the 5th and 6th cervical roots merging to become the superior trunk. The first site for the CSA measurement of the SSN was where it branched from the posterior aspect of the superior trunk (Figure 1A). The nerve was tracked further laterally, passing the suprascapular notch to reach the anterior edge of the suprascapular fossa, where the second CSA measurement was taken (Figure 1B). Finally, we placed the transducer parallel and caudal to the scapular spine to image the third site of the CSA measurement inside the spinoglenoid notch (Figure 1C; Wu et al., 2018).

The CSA measurements were then conducted by another specialist who was blinded to the diagnoses and clinical presentations of the participants. The function of freehand selection of the image processing software, Image J (National Institutes of Health, Rockville Pike, Bethesda, MD, United States), was used to outline the nerve border and to estimate its CSA. For the CSA measurement of the most proximal section of the SSN, we took the nerve fascicles inside the hyperechoic epineurium into account (Figure 1A). To measure the CSA of the segments over the supraspinatus and infraspinatus fossa, the epineurium was difficult to differentiate from the fascicles due to the limited US resolution in the deep region. Therefore, we measured the entire nerve CSA, which included the epineurium (Figures 1B,C). According to our previous study, the intra-rater reliability (quantified by the intra-class correlation coefficient) of measuring the CSA of the SSN inside the epineurium ranged between 0.853 and 0.884, whereas the corresponding inter-rater reliability ranged from 0.749 to 0.785 (Wu et al., 2018). The standard error of measurement of the same approach for measuring the CSA of the SSN at the brachial plexus ranged between 0.20 and 0.30 mm^2^.

### Sample Size Estimation and Statistical Analysis

The sample size calculation was based on an antecedent study (Wu et al., 2018) using a similar method to measure the CSA of the SSN. In the aforementioned study, US imaging showed a between-gender difference of approximately 0.5 mm^2^ in the CSA of the SSN at the brachial plexus level, with a standard deviation of nearly 0.6 mm^2^. Therefore, the sample size in our study was designed to recognize a CSA difference of 0.5 mm^2^ between the groups with a standard deviation (SD) of 0.6 mm^2^. The alpha level (α) and the power (β) of the study were set at 5 and 80%, respectively. The minimum required sample size was 46 persons.

The data is presented as median (minimum, maximum), frequency, or percentage. For the univariate analysis, one-way analysis of variance (ANOVA) was used to compare the normally distributed continuous variables across different groups with the use of Bonferroni corrections for multiple comparisons. The Shapiro–Wilk test was used to examine data normality. The Kruskal–Wallis test was employed instead if the data were not normally distributed. To maintain the assumption of independence for ANOVA, group comparisons regarding nerve CSA were based on the unit of an individual person by averaging the values from bilateral sides (Menz, 2004). Categorical variables were analyzed using the chi-square test or Fisher’s exact test (in case of sparse data). Paired continuous and categorical data (e.g., observations and measurements at the non-painful and painful sides in patients with unilateral shoulder pain) were analyzed using the paired *t*-test (or Wilcoxon signed-rank test in case of non-normality of the data) and McNemar’s test, respectively.

The generalized estimation equation (GEE) model was used to investigate the association between the CSA of the SSN and various rotator cuff tendon abnormalities. The GEE was suitable to deal with the auto-correlated data (Zorn, 2001), such as the nerve CSA from the right or the left shoulder of the same participant. The dependent variables in the GEE model were the CSAs of the SSN at the brachial plexus, supraspinatus fossa, and infraspinatus fossa. The independent variables included age, sex, shoulder laterality, group and various kinds of shoulder tendon pathologies (i.e., biceps tendinopathy, biceps tendon tear, subscapularis tendinopathy, subscapularis tendon tear, supraspinatus tendinopathy, supraspinatus tendon partial-thickness tear, supraspinatus tendon full-thickness tear, infraspinatus tendinopathy, and infraspinatus tendon tear). Shoulder laterality was treated as a within-subject factor. As no pilot knowledge for its covariance matrix was available, the covariance structure we chose was unstructured. Regarding *post hoc* analysis, the Bonferroni method was selected to correct multiple comparisons when displaying estimated marginal means. Furthermore, the goodness-of-fit of the model was assessed using the Corrected Quasi-likelihood under Independence Model Criterion (QICC) (Pan, 2001). The analyses were carried out using MedCalc 14.0 (MedCalc Software, Ostend, Belgium) and SPSS 21.0 (IBM SPSS Statistics for Windows, Version 21.0, Armonk, NY, United States). A *p*-value < 0.05 was considered statistically significant.

## Results

**FIGURE 2:**
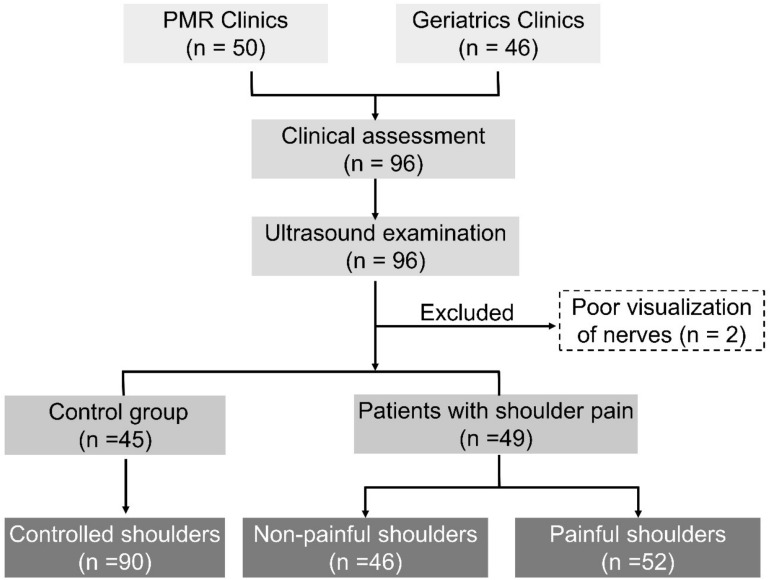
Flow diagram of participant recruitment.

A total of 96 participants were initially enrolled in the clinical and US examinations. Two patients were excluded due to difficulty in visualizing their SSN inside the supraspinatus fossa. Among the remaining 94 participants, 45 were classified into the control group (with bilaterally asymptomatic shoulders) and 49 were classified into the group with shoulder pain (with at least one painful shoulder) (Figure 2). In our study, the presence of shoulder pain was defined as a total SPADI score >0. The CSA values of the SSN were similar (control vs. shoulder pain groups) at the level of the brachial plexus, supraspinatus fossa, and infraspinatus fossa (Table 1). The number and proportion of positive physical and US findings in both groups are presented in Supplementary Table 1.

**TABLE 1:** Subject characteristics and suprascapular nerve (SSN) cross-sectional area measurements of the study participants.

	Control group (*n* = 45)	Patients with shoulder pain (*n* = 49)	*p*-value
**Demographics**			
Age (year)	67(51,80)	67(53,81)	0.432^a^
Female gender (number,%)	18(40.00%)	26(53.06%)	0.205^b^
Height (cm)	160.00(148.00,176.00)	160.00(150.00,179.00)	0.570^a^
Weight (kg)	58.50(42.00,82.3)	63.00(44.40,85.00)	0.087^a^
Body mass index (kg/m^2^)	23.11(17.48,28.47)	24.02(15.49,28.93)	0.069^a^
**Clinical presentation**			
Unilateral shoulder pain (number,%)	0(0.00%)	46(93.88%)	<0.001*^c^
Bilateral shoulder pain (number,%)	0(0.00%)	3(6.12%)	0.243^c^
Pain duration (month)	0.00(0.00,0.00)	3.00(0.03,84.00)	<0.001*^d^
**Average cross-sectional area of SSN**			
At the brachial plexus level (mm^2^)	1.57(0.96,2.55)	1.57(1.11,2.04)	0.252^d^
At the supraspinatus fossa (mm^2^)	8.80(4.83,11.90)	8.43(5.01,15.59)	0.806^d^
At the infraspinatus fossa (mm^2^)	9.03(4.79,12.97)	7.87(4.38,13.09)	0.594^a^

Values are given as median (minimum, maximum). *P*-values pertain to between-group comparisons. * Indicates *p* < 0.05. ^*a*^Indicates that the comparisons are performed by one-way ANOVA. ^*b*^Indicates that the comparisons are performed by the chi-square test. ^*c*^Indicates that the comparisons are performed by Fisher’s exact test. ^*d*^Indicates that the comparisons are performed by Kruskal–Wallis test.

In the group with shoulder pain, only three participants had bilateral shoulder pain, and the rest (*n* = 46) presented with unilateral shoulder pain. Of all the painful shoulders (*n* = 52), 67.3% were on the left side. The median total SPADI score was 28.84, and the corresponding median VAS scores ranged from 5 (pain at rest or at night) to 6 (pain during overhead activity).

We further analyzed the data from 46 patients with unilateral shoulder pain. Regarding the CSA of the SSN, the univariate analysis revealed no significant difference between the painful and non-painful shoulders at the brachial plexus level. At the level of the supraspinatus and infraspinatus fossa, the SSN was significantly larger in the painful shoulders than in the asymptomatic shoulders (Table 2). Likewise, rotator cuff tendon pathologies were more frequent in the painful shoulders than in the asymptomatic shoulders (Table 2).

**TABLE 2:** Suprascapular nerve (SSN) cross-sectional area measurements and ultrasound findings in subjects with unilateral involvement.

	Non-painful shoulder (*n* = 46)	Painful shoulder (*n* = 46)	*p*-value
**Cross-sectional area of SSN**			
At the brachial plexus level (mm^2^)	1.52(1.07,2.17)	1.51(1.12,2.48)	0.918^a^
At the supraspinatus fossa (mm^2^)	6.82(4.65,15.04)	9.45(4.87,15.06)	< 0.001^*a^
At the infraspinatus fossa (mm^2^)	7.21(4.73,12.83)	8.71(4.03,14.71)	< 0.001^*a^
**Positive ultrasound finding**			
Biceps tenosynovitis (number, %)	1(2.17%)	6(13.04%)	< 0.001^*b^
Biceps tendon tear (number, %)	0(0.00%)	1(2.17%)	< 0.001^*b^
Subscapularis tendinopathy (number, %)	12(26.08%)	16(34.78%)	0.008^*b^
Subscapularis tendon tear (number, %)	4(8.69%)	8(17.39%)	< 0.001^*b^
Supraspinatus tendinopathy (number, %)	17(36.95%)	35(76.08%)	0.345^b^
Supraspinatus partial thickness tear (number, %)	6(13.04%)	6(13.04%)	< 0.001^*b^
Supraspinatus full-thickness tear (number, %)	1(2.17%)	11(23.91%)	< 0.001^*b^
Infraspinatus tendinopathy (number, %)	12(26.08%)	19(41.30%)	0.024^*b^
Infraspinatus tendon tear (number,%)	1(2.17%)	4(8.69%)	< 0.001^*b^

Values are given as median (minimum, maximum). *P*-values pertain to between-group comparisons. * Indicates *p* < 0.05. ^*a*^Indicates that the comparisons are performed by Wilcoxon signed-rank test. ^*b*^Indicates that the comparisons are performed by McNemar test.

**FIGURE 3:**
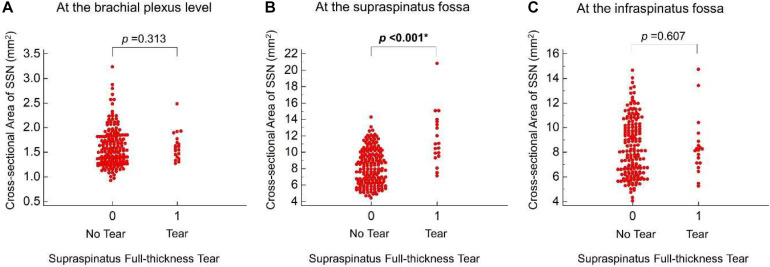
Scatter plots of the cross-sectional area of the suprascapular nerve between shoulders with and without supraspinatus full-thickness tear near the brachial plexus **(A)**, at the supraspinatus fossa **(B)**, and at the infraspinatus fossa **(C)**. *P*-values pertain to β coefficients of the generalized estimating equation analysis. * Indicates statistical significance.

Regarding the GEE analysis, female sex was associated with a decrease in the CSA of the SSN at the brachial plexus level (β coefficient = −0.269 mm^2^, *p* < 0.001). For rotator cuff lesions, a significant positive association (β coefficient = 4.068 mm^2^, *p* < 0.001) was identified between the full-thickness tear of the supraspinatus tendon and the CSA of the SSN at the supraspinatus fossa (Table 3). No significant association was identified between the partial-thickness tear of the supraspinatus tendon and the CSA values of the SSN at the three measured sites. The CSA values of the SSN in shoulders with and without supraspinatus tendon full-thickness tears are shown in Figure 3. A sensitivity analysis was performed by excluding an outlier of the CSA at the supraspinatus fossa. The positive association between the nerve CSA and supraspinatus tendon full-thickness tear remained significant (β coefficient: 3.517 mm^2^, *p* < 0.001). As supraspinatus tendon full-thickness tear was the only variable significantly predicting SSN enlargement at the supraspinatus fossa, we also tested which model (with or without the addition of supraspinatus tendon full-thickness tear) yielded higher goodness-of-fit. The QICC value without the variable of supraspinatus tendon full-thickness tear was 1106.462, whereas that with supraspinatus tendon full-thickness tear was 929.757, indicating that the latter was better.

**TABLE 3:** Associations among suprascapular nerve cross-sectional area measurements and the clinical characteristics (expressed by β coefficients and their 95% confidence intervals).

	At brachial plexus	At supraspinatus fossa	At infraspinatus fossa
Age (years)	−0.003 (−0.01 to 0.005)	0.001 (−0.04 to 0.04)	−0.021 (−0.07 to 0.03)
	*p* = 0.460	*p* = 0.952	*p* = 0.494
Female gender	−0.269 (−0.39 to −0.14)	−0.600 (−1.39 to 0.18)	−0.703 (−0.10 to 1.51)
	*p* < 0.001*	*p* = 0.136	*p* = 0.088
Left side	0.052 (−0.03 to 0.14)	−0.375 (−0.85 to 0.10)	−0.410 (−0.85 to 0.03)
	*p* = 0.247	*p* = 0.127	*p* = 0.072
Group^*a*^	−0.129 (−0.28 to 0.02)	−0.449 (−1.33 to 0.43)	−0.393 (−1.27 to 0.49)
	*p* = 0.091	*p* = 0.319	*p* = 0.384
Biceps tendinopathy	−0.064 (−0.27 to 0.14)	0.081 (−1.77 to 1.93)	−0.534 (−2.17 to 1.10)
	*p* = 0.550	*p* = 0.932	*p* = 0.522
Biceps tendon tear	0.052 (−0.22 to 0.33)	−1.688 (−4.64 to 1.26)	−0.696 (−2.13 to 0.74)
	*p* = 0.716	*p* = 0.263	*p* = 0.342
Subscapularis tendinopathy	−0.008 (−0.11 to 0.09)	−0.540 (−1.34 to 0.26)	−0.608 (−1.41 to 0.19)
	*p* = 0.880	*p* = 0.190	*p* = 0.138
Subscapularis tear	0.068 (−0.14 to 0.27)	−0.803 (−1.99 to 0.38)	0.378 (−1.04 to 1.80)
	*p* = 0.520	*p* = 0.187	*p* = 0.603
Supraspinatus tendinopathy	−0.045 (−0.16 to 0.07)	−0.307 (−1.13 to 0.518)	−0.496 (−1.32 to 0.33)
	*p* = 0.465	*p* = 0.466	*p* = 0.242
Supraspinatus partial tear	0.020 (−0.16 to 0.20)	−0.791 (−1.84 to 0.26)	−0.415 (−1.38 to 0.55)
	*p* = 0.832	*p* = 0.141	*p* = 0.402
Supraspinatus full thickness tear	0.120 (−0.11 to 0.35)	4.068 (2.10 to 6.03)	0.403 (−1.13 to 1.93)
	*p* = 0.313	*p* < 0.001*	*p* = 0.607
Infraspinatus tendinopathy	−0.032 (−0.17 to 0.11)	0.811 (−0.32 to 1.94)	0.886 (−0.15 to1.92)
	*p* = 0.655	*p* = 0.162	*p* = 0.096
Infraspinatus tear	−0.003 (−0.22 to 0.21)	−1.244 (−3.18 to 0.69)	−1.248 (−2.76 to 0.27)
	*p* = 0.977	*p* = 0.209	*p* = 0.107

^*a*^Indicates that the control group is taken as the reference. * Indicates *p* < 0.05.

## Discussion

The present study yielded two main findings for the older population. First, in patients with unilateral shoulder pain, the SSN seems to be enlarged distally (but not proximally) in the painful shoulders. Second, an independent association was identified between the full-thickness tears of the supraspinatus tendon and swelling of the SSN in the supraspinatus fossa.

In patients with unilateral shoulder pain, the CSA of the distal SSN was larger in the painful shoulders. As enlargement of the nerve fascicle is a common presentation of entrapment neuropathy (Chang et al., 2020b), a significant association between shoulder pain and abnormal nerve morphology could be made. Our findings also implied that the entrapped segment might have occurred distal to the suprascapular notch. Furthermore, there was a higher prevalence of rotator cuff pathologies in the painful than in the non-painful shoulders in patients with unilateral involvement. Because we only included elderly patients, there was a chance that they actually had cervical degenerative spine disease with pain radiating to the shoulder regions (Theodore, 2020). Based on our observation, we speculated that the aforementioned concern had minimal impact on our patient population as the majority of painful shoulders did reveal pathologies identified by US imaging.

In our GEE analysis, female sex was associated with a decrease in the CSA of the SSN at the brachial plexus level. This finding was compatible with a previous similar study (Wu et al., 2018), which reported that women (vs. men) had smaller cervical nerve roots and SSN proximally. We also found that the SSN appeared enlarged at the supraspinatus fossa in our older population with full-thickness tears of the supraspinatus tendon (vs. those without). However, the CSA difference was not observed either at the level of the brachial plexus or at the infraspinatus fossa. Increase in the nerve size is a common consequence of entrapment neuropathy and is more likely to occur in the region proximal to the site of compression (Raz et al., 2015). This phenomenon is attributed to the obstruction of the axoplasmic flow after compression of the distal nerve fascicles.

**FIGURE 4:**
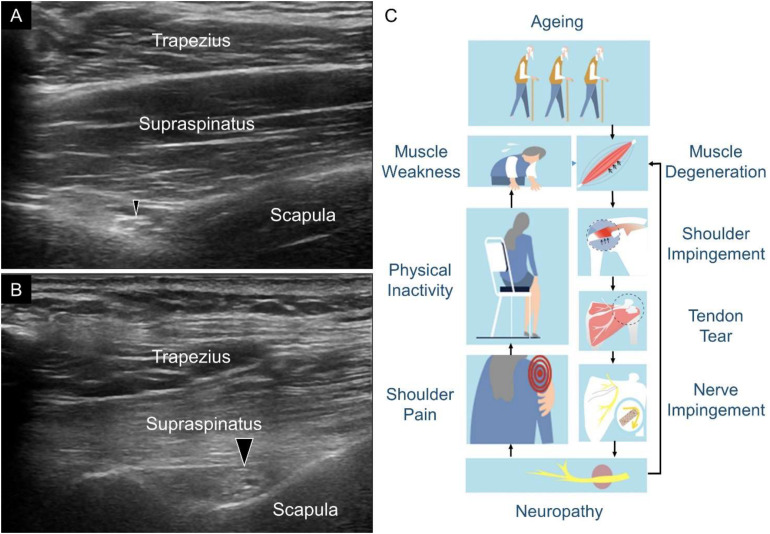
Ultrasound imaging of the suprascapular nerve (SSN) (black arrowheads) in one shoulder without supraspinatus tendon full-thickness tear **(A)** and the contralateral shoulder with supraspinatus full-thickness tear **(B)**. Atrophy of the supraspinatus muscle and enlargement of the SSN with thickened epineurium were seen in the shoulder with supraspinatus tendon full-thickness tear. A plausible flow diagram demonstrates how rotator cuff tendon tears interplay with SSN abnormalities in the aging population **(C)**.

The SSN is vulnerable to entrapment in the tunnel connecting the supraspinatus and infraspinatus fossa. A recent cadaveric study investigating the embalmed shoulders delineated the course of the SSN and found a sharp angle between the segments proximal and distal to the spinoglenoid notch (Fabis-Strobin et al., 2018). A decrease in this angle would increase the traction force pulling the SSN against the lateral edge of the scapular spine. In patients with a large-sized supraspinatus or infraspinatus tendon tear, the retracted tendon fibers would bring the SSN more medially (Shi et al., 2012), making the angle more steep and a subsequent traction injury more likely. This possibility could explain why we visualized the enlargement of the SSN at the supraspinatus fossa (Figures 4A,B).

A vicious cycle between SSN abnormality and shoulder pain might occur in the older population (Figure 4C). First, SSN dysfunction can lead to shoulder pain and subsequent physical inactivity. A sedentary lifestyle can cause further weakness of the shoulder girdle muscles. Second, SSN neuropathy can aggravate age-related rotator cuff tendon degeneration, which was evident in the animal model (Gereli et al., 2020). Both of these factors can increase the risk of rotator cuff tendon tears. Once the extent of the rotator cuff tendon tears reaches a certain severity, the SSN is again entrapped, which leads to further worsening of the symptoms. To this end, we believe that the findings of the present study have important clinical implications. In older adults with shoulder pain, a systematic US assessment should be performed to scrutinize rotator cuff tendon pathologies. If a full-thickness tear of the supraspinatus tendon with recalcitrant pain is identified, concomitant SSN entrapment can be considered. For symptomatic relief, US-guided perineural hydrodilatation can easily be conducted as the next step in the management (Chang et al., 2020b).

In the present study, we also performed various physical tests on our participants. Older adults were our target population, and many older adults have been reported to have cervical spine diseases with pain radiating to the shoulders (Theodore, 2020). The physical tests enabled us to locate the actual pain origin. However, according to a recent review (Chang et al., 2020a), the accuracy of physical tests to diagnose shoulder pathologies was unsatisfactory. Hence we only chose shoulder US, a more reliable assessment, to analyze the CSA of the SSN.

Some limitations of this study need to be acknowledged. First, our study used a cross-sectional design. The causal relationship between rotator cuff tendon tears and SSN abnormalities requires a prospective cohort study for further evaluation. Second, the cause of rotator cuff tear is usually assumed to be tendon degeneration in the older people and repetitive trauma in the younger population. As this study only focused on people older than 60 years, the generalizability of our findings to other age groups might be limited. Third, the pathological changes observed in the US examinations might have been attributed to “normal” aging-related degeneration. A previous observational study demonstrated thicker and more hypoechoic supraspinatus tendons in asymptomatic elderly participants than in younger participants (Yu et al., 2012). However, since no middle-aged or young adults were enrolled in our study, we were not able to test this hypothesis.

## Conclusion

In older adults, shoulder pain might be related to abnormalities of the distal SSN. Among various rotator cuff lesions, only the full-thickness tear of the supraspinatus tendon appears to be independently associated with SSN enlargement at the supraspinatus fossa. In the case of the patients having large rotator cuff tendon tears that are poorly responsive to conservative treatments, SSN entrapment should be considered and managed accordingly.

## Data Availability Statement

The original contributions presented in the study are included in the article/Supplementary Material, further inquiries can be directed to the corresponding author/s.

## Ethics Statement

The studies involving human participants were reviewed and approved by National Taiwan University Hospital (IRB No. 20180405RIND). The patients/participants provided their written informed consent to participate in this study.

## Author Contributions

K-VC, W-TW, L-RC, and H-CC: the study concept and design, or analysis and interpretation of data. W-TW, LÖ, and K-VC: drafting/revising the manuscript for important intellectual content. All authors approval of the final version to be published.

## Conflict of Interest

The authors declare that the research was conducted in the absence of any commercial or financial relationships that could be construed as a potential conflict of interest.

## Abbreviations

SSN: suprascapular nerve
US: ultrasound
CSA: cross-sectional area
SPADI: Chinese version of the Shoulder Pain and Disability Index
VAS: visual analog scale
GEE: generalized estimation equation.
