# Supplementary material for: Quantitative Ultrasonographic Analysis of Changes of the Suprascapular Nerve in the Aging Population With Shoulder Pain
Source: Front Bioeng Biotechnol. 2021 Feb 19;9:640747. doi: 10.3389/fbioe.2021.640747 (PMC7933457; doi:10.3389/fbioe.2021.640747)
Supplement: Supplementary file 1 [file Table_1.DOCX]

| **Supplementary Table 1.** Physical and ultrasound findings of the shoulders in the control group and patients with shoulder pain | | | |
| --- | --- | --- | --- |
|  | Control group | Patients with shoulder pain | |
|  | All shoulders (n=90) | Non-painful shoulders (n=46) | Painful shoulders (n=52) |
| Positive physical findings | | | |
| Bicipital groove tenderness (number, %) | 0 (0.00%) | 0 (0.00%) | 27 (51.92%) |
| Speed test (number, %) | 0 (0.00%) | 0 (0.00%) | 24 (46.15%) |
| Yergason’s test (number, %) | 0 (0.00%) | 0 (0.00%) | 18 (34.62%) |
| Empty can test (number, %) | 0 (0.00%) | 0 (0.00%) | 32 (61.54%) |
| Neer test (number, %) | 0 (0.00%) | 0 (0.00%) | 32 (61.54%) |
| Hawkins-Kennedy test (number, %) | 0 (0.00%) | 0 (0.00%) | 27 (51.92%) |
| Painful arc test (number, %) | 0 (0.00%) | 0 (0.00%) | 30 (57.69%) |
| Positive ultrasound findings | | | |
| Biceps tenosynovitis (number, %) | 8 (8.88%) | 1 (2.17%) | 7 (13.46%) |
| Biceps tendon tear (number, %) | 1 (1.11%) | 0 (0.00%) | 3 (5.76%) |
| Subscapularis tendinopathy (number, %) | 13 (14.44%) | 12 (26.09%) | 17 (32.69%) |
| Subscapularis tendon tear (number, %) | 1 (1.11%) | 4 (8.69%) | 9 (17.31%) |
| Supraspinatus tendinopathy (number, %) | 15 (16.67%) | 17 (36.96%) | 41 (78.85%) |
| Supraspinatus partial thickness tear (number, %) | 7 (7.77%) | 6 (13.04%) | 7 (13.46%) |
| Supraspinatus full-thickness tear (number, %) | 2 (2.22%) | 1 (2.17%) | 16 (30.77%) |
| Infraspinatus tendinopathy (number, %) | 0 (0.00%) | 12 (26.09%) | 23 (44.23%) |
| Infraspinatus tendon tear (number, %) | 0 (0.00%) | 1 (2.17%) | 4 (7.69%) |
| Values are given as number and percentage. | | | |
